# Decision Making in Pediatric Heart Failure and Transplant: A Qualitative Analysis of Parental Decision Making

**DOI:** 10.1111/petr.70229

**Published:** 2025-11-21

**Authors:** Kari A. Phillips, Elizabeth Lancaster, Stephanie Tuckett, Patrick Galyean, Susan L. Zickmund, Kimberly M. Molina, Nelangi M. Pinto, Elissa M. Ozanne

**Affiliations:** ^1^ Division of Pediatric Cardiology, Department of Pediatrics University of Utah Salt Lake City Utah USA; ^2^ Division of Critical Care Medicine, Department of Pediatrics University of Utah Salt Lake City Utah USA; ^3^ Division of Epidemiology, Department of Internal Medicine, Qualitative Research Core University of Utah Salt Lake City Utah USA; ^4^ Informatics, Decision‐Enhancement and Analytic Sciences Center Veterans Affairs Salt Lake City Healthcare System Salt Lake City Utah USA; ^5^ Division of Epidemiology, Department of Internal Medicine University of Utah School of Medicine Salt Lake City Utah USA; ^6^ Division of Pediatric Cardiology, Department of Pediatrics Dartmouth Geisel School of Medicine Hanover New Hampshire USA; ^7^ Division of Pediatric Cardiology, Department of Pediatrics Seattle Children's Hospital/University of Washington Seattle Washington USA; ^8^ Department of Population Health Sciences University of Utah Salt Lake City Utah USA

**Keywords:** family centered care, medical decision making, patient centered care, pediatric heart failure, pediatric heart transplantation, surrogate medical decision making

## Abstract

**Background:**

Parents of children with advanced heart failure face complex, emotionally charged decisions regarding mechanical circulatory support (MCS) and heart transplantation. Understanding their informational needs and decision‐making factors is crucial to optimizing their education and providing support.

**Methods:**

We conducted a qualitative study with parents of children (< 18 years) who are listed for or who underwent heart transplantation within the past 3 years. Participants engaged in focus groups or interviews exploring informational needs, decision‐making perceptions, influencing factors, and educational tool preferences. Transcripts underwent thematic coding and analysis.

**Results:**

Nineteen parents participated. Four major themes emerged: (1) Informational Needs—Parents sought medical facts from providers and valued lived experiences from like‐peers for practical insights. (2) Decision Making Perception—Most viewed transplant as the only viable option; others viewed it as a genuine decision; all desired autonomy in decision making. (3) Decision Factors—Survival and quality of life were primary drivers; external stressors (financial, logistical, emotional) were seen as inevitable consequences rather than determinants. (4) Parental Distress—Emotional burden was substantial, with parents emphasizing the need for better preparation and mental health support. Parents preferred a multimodal educational tool incorporating digital and physical resources, visual aids, and customizable journey maps.

**Conclusions:**

These findings highlight the significance of peer support groups, the role of social support in managing external stressors, the potential of decision support tools for families to clarify their goals.

## Graphical abstract

After engaging with 19 parents of children who had undergone or are listed for heart transplant, we concluded that survival and quality of life primarily drive their medical decision making. They require like‐peer education, extensive emotional/social support, and would benefit from decision support tools to clarify their goals.

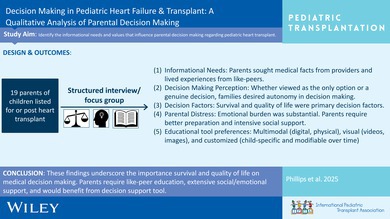

**Abbreviations**
ECMO: extracorporeal membrane oxygenation
MCS: mechanical circulatory support
VAD: ventricular assist device

## 1 Introduction

Pediatric heart failure is a chronic disease with no definitive cure and high morbidity and mortality [1]. Many patients with heart failure require escalation of medical care as their disease progresses and impacts other organ systems. Treatment for end‐stage pediatric heart failure can entail intensive therapies such as mechanical circulatory support (MCS), invasive respiratory support, and renal replacement therapy, as well as heart transplantation. For families, such treatments involve considerable financial investment [2, 3] and can disrupt family dynamics [4], parental employment [3], and psychosocial support networks significantly due to prolonged hospitalizations and potential geographic relocation for proximity to a transplant center [5]. The immense medical and psychosocial burden has the potential to negatively impact the short‐ and long‐term quality of life and mental health for both the patient and their entire family [6, 7, 8].

The chronic medical burden and morbidity associated with intensive heart failure treatments necessitate complex, emotionally charged, and value‐laden decision making, particularly to employ MCS (i.e., ventricular assist device [VAD] or extracorporeal membrane oxygenation [ECMO]) and/or list for heart transplant. Parents (with or without input from the patient) must decide the best options on behalf of their child and their family. While this decision making is immensely stressful, caregivers desire explicit involvement in the decision to pursue heart transplant [9, 10].

Pediatric transplant programs are required to employ formal education for families who are considering intensive therapies (particularly MCS) or heart transplant. To optimize educational and decision support tools for families faced with these complex decisions, it is critical to understand the informational needs and the underlying drivers that guide decision making in the context of pediatric heart transplant [11]. While it is clear that the amount and type of information provided to families impacts decision making [12], there is scarce literature exploring the informational needs of families faced with the decision to pursue advanced heart failure therapies. In addition to information, several other domains have been identified as important to parental medical decision making in other contexts (end of life, children with developmental delay and medical complexity, premature infants, complex congenital heart disease) including parental education, coping strategies, and religious background, the child's prognosis and previous medical needs/interventions, and the child's quality of life and best interest [12, 13, 14]. How these factors specifically influence parental decision making has not been examined in pediatric heart failure populations.

The objective of this qualitative study was to identify the informational needs as well as factors and values that influence parental decision making regarding intensive therapies and heart transplant for children with advanced heart failure. We also sought parental preferences for features and formatting of an educational tool or decision aid that could be used in this context.

## 2 Patients and Methods

### 2.1 Participants

For this cross‐sectional, qualitative study, we recruited parents of pediatric patients (< 18 years old at the time of transplant listing) who underwent heart transplantation or were currently listed for heart transplantation at Primary Children's Hospital. Participants were identified via our institution's heart transplant database and wait list. Eligible participants were contacted via letter or email soliciting interest with a subsequent phone call or in‐person follow‐up. Only parents of children who were listed for or had undergone transplantation in the last 3 years prior to the study were included. The three‐year timeframe was selected to optimize parents' retrospective recall of their decision‐making in the peri‐transplant timeframe. Families pre‐ and post‐transplant were included to elicit both perspectives of those in the midst of decision‐making and who are reflecting on decisions amidst post‐transplant realities. For this study, “parent” was defined as a person 18 years of age or older who had legal guardianship, medical decision‐making authority, and an active caregiving role over the child at the time of listing and/or transplantation. Eligible participants were English‐speaking. Parents were eligible regardless of their child's vital status and listing status at the time of recruitment. Participants were provided a consent form via email which was reviewed at the start of the focus group or interview; they provided verbal consent to participate.

### 2.2 Data Collection

Participants engaged in either a focus group discussion (post‐transplant families, 2 h) or a structured interview (pre‐transplant families, 30–45 min). All interviews were conducted by the University of Utah Community Collaboration and Engagement Team, a Clinical and Translational Science Institute supported group specializing in focus groups and interviews across disciplines. Pre‐transplant families were engaged via structured interviews (as opposed to focus groups) to avoid distress or undue influence in hearing the decision making priorities of others in the midst of their own ongoing medical decision making. The conversation guide (identical for focus groups and interviews) was developed using a literature review [10, 11, 14] and guidance from clinician experts, decision scientists, qualitative research experts, and participant engagement experts. Finalized content included: (1) key information caregivers desired/required to engage in informed decision making, (2) outcomes and goals of care integral to this decision, (3) non‐medical factors influencing decision making, and (4) preferences for educational tools and decision aids. Using the conversation guide, discussions were conducted by trained members of the research team. They were audiotaped and transcribed verbatim. Focus groups and interviews continued until thematic saturation was achieved. All study procedures were approved by the University of Utah and Primary Children's Hospital Institutional Review Boards.

### 2.3 Analysis

Qualitative analysis of the transcripts was completed by The University of Utah Qualitative Research Core (led by author SZ), a Clinical and Translational Science Institute supported group specializing in qualitative analysis and mixed methods research. They used a standardized five‐stage method using the Editing Approach Crabtree and Miller methodology which is an inductive qualitative method based on tenets from grounded theory and is developed to be specific to the context of research conducted in the health sciences. The method includes five steps: familiarization with the raw data, identification of categories and themes, data indexing, charting and mapping, and interpretation using representative quotation [15]. We were also guided by the PCORI qualitative standards for shaping research questions and guiding the process [16, 17]. The software program Atlas.ti was used to manage all qualitative data. All transcripts were de‐identified. A codebook was developed with specific inclusion/exclusion criteria with the qualitative analysts working with the larger study team. Quotations were captured and categorized within the codebook. Representative quotations were captured verbatim. Authors EL (MSc) and ST (MSc), under the supervision of SZ (PhD), co‐coded (with validation of inter‐coder reliability) both focus groups and interviews with the coders using an adjudication process to resolve differences. Data analysts were qualitative research and analysis experts but did not work clinically in pediatric heart failure and transplant, thus minimizing personal bias in coding and theme identification. Codes and themes were reviewed by clinicians with shared decision‐making and pediatric heart failure and transplant experience to validate findings and provide context and relevance.

## 3 Results

### 3.1 Participants

Of 25 parents contacted via phone or in person (after 41 were pre‐notified via letter/email), 19 consented to participate and completed either a focus group (nine participants, post‐transplant) or an interview (10 participants, pre‐transplant). Of note, one family whose child had died (pre‐transplant) initially agreed to participate but subsequently withdrew citing an unrelated family emergency/stressor. Three other families of deceased children could not be reached despite multiple phone call attempts. Most participants were female (17, 89%), of White race (13, 68%), and Christian (13, 68%) (Table 1). The underlying condition in participants' children was congenital heart disease (13, 68%), cardiomyopathy (5, 26%), and acquired heart disease (1, 5%). Twelve (63%) children were supported on VADs. The average age of participants' children at the time of transplant listing was 5 years (range 1 month–16 years). All children of participants were alive at the time of study participation.

**TABLE 1:** Demographic characteristics of parent participants (*n* = 19).

Demographic characteristic	*N* (%)
Age (average, range)	34.8 (26–49) years
Gender identity	
Female	17 (89%)
Male	2 (11%)
Race	
White	13 (68%)
Latino/Latina	3 (16%)
More than one	1 (5%)
Sexual orientation	
Heterosexual	18 (95%)
Prefer not to answer	1 (5%)
Employment status (at time of child's transplant listing)	
Employed	12 (63%)
Unemployed	7 (37%)
Annual household income	
$10 000–$24 999	1 (5%)
$25 000 ‐ $49 999	2 (11%)
$50 000 ‐ $74 999	5 (26%)
$75 000 or more	10 (53%)
Prefer not to answer	1 (5%)
Religious affiliation	
Christian	13 (68%)
Atheist	1 (5%)
Agnostic	2 (11%)
No affiliation	3 (16%)
Highest level of education	
Less than high school	1 (5%)
High school	1 (5%)
Some college	7 (37%)
Associate's degree	2 (11%)
Bachelor's degree	2 (11%)
Master's degree	5 (26%)
Neighborhood type	
Urban	7 (37%)
Suburban	11 (58%)
Prefer not to answer	1 (5%)
Relationship status (at the time of child's transplant listing)	
Married	18 (95%)
Single (never married)	1 (5%)
Children in household (at the time of child's transplant listing)	
1–2	9 (47%)
3–4	8 (42%)
5+	2 (11%)
Child's underlying cardiac diagnosis	
Congenital heart disease	13 (68%)
Cardiomyopathy	5 (26%)
Acquired heart disease	1 (5%)
Child on ventricular assist device support before transplant	
Yes	12 (63%)
No	7 (37%)

### 3.2 Themes

Qualitative data analysis revealed four themes. Three themes aligned with the conversation guide content: informational needs, decision‐making perception, and decision factors. An additional theme emerged spontaneously: parental distress. The themes are outlined below and major sub‐themes within each are described. Representative quotations are provided (Table 2). Several preferences for educational tools and decision aids were also revealed and outlined below (quotations not included).

**TABLE 2:** Representative quotations for seven sub‐themes within four themes: informational needs, decision‐making perception, decision factors, and parental distress.

	Sub‐theme	Representative quotations
Informational needs	Basic medical information	“I think the thing that really helped me was all the immediate education that [hospital] provided in their transplant program, like the binder with all the resources and all the facts and a lot of the statistics” “I think also knowing the side effects, like just because you're going to have a heart transplant, we're fixing one problem, but then you're welcoming a lot, with side effects, medications, and all of those things” “My wife and I didn't know until we started down this process…is when you get a heart transplant, it's not a one and done. You will come back. And so…understanding that timeline”
	Practical advice	4 “…so just kind of asking [other families] sort of what their issues and other things have been. And so that's really where a lot of that non‐professional knowledge came from, people who have experienced it” 5 “They got permission from another family that was at the hospital, and we were able to come see them and their little baby, and they were able to tell us a little bit more about their experience and what it was like for them, and how her quality of life is now, you know?” 6 “So I do feel like a lot of my knowledge actually came from other patients. And like, their experiences”
Decision making perception	Foregone conclusion versus decision	7 “I mean, it kind of was like an easy decision, because it was like either she's going to die, or you can get a heart transplant. And we're like: Well, we want to do what we can to give her the longest life possible. And so that's how we kind of decided to do transplant” 8 “We really wanted to weigh the options of whether or not it was something that we wanted to put her through” 9 “So it was more just on the two of us with the medical team, to kind of: Is this the right path forward? Will it be comfortable? And then, will the outcome be worth the kind of process we would put him through?”
	Decision making role	10 “But my opinion would say: Present the best and present the worst, and then let families make their decision from that” 11 “I wanted 100% of the role to make the decision” 12 “When we were going through it, we really trusted our doctors a lot, and sought their opinions, and we also talked about it. And they asked us an important question, they wanted us to think about the best quality of life for our baby…And so they kind of left that open for us to decide”
Decision factors	Survival and quality of life	13 “I think that would be the biggest deal breakers, is if the quality of life for her was just not good” 14 “Because yes, science and medicine can do wonderful things, as we've already been attested to…. So it's just making sure that he would be able to be a normal kid and live a kid life, and that this wouldn't hold him back, indefinitely, to a point where he's not able to enjoy the life that we were fighting for” 15 “But the big questions we asked were: What would he achieve, quality of life‐wise? Like, would there be any limitations?”
	Non medical factors	16 “We were kinda worried about some of the…process after the transplant…, and then also the effect it would have on our family. Like, not being able to travel as much, or travel as far. Those were definitely some things we had to take into consideration” 17 “…we felt like it was our opportunity to provide her with…every opportunity to survive. And so we just kind of felt like…we just need to accept…whatever changes it will cause to our family, like we will need to adapt” 18 “This is my child, I'll do anything that will help her”
Parental distress		19 “I just wish that there was like, more support for parents' mental health, and like, therapists that had experience in this kind of situation that would be readily available to parents. Or even require parents to go through like, some therapy classes, or something like that, for coping and helping their child cope” 20 “Like, my mental health is struggling, personally…And it just puts a lot of like, weight on my shoulders, because I have full custody of the kids, and now I have all the stress of [child's name], and all of his medicines, and all of his treatments, and all of his doctor's appointments and bloodwork, is all of my responsibility” 21 “I just think it would have offered a little bit more understanding as to what kind of emotional turmoil you would be going through” 22 “It's painful to see him suffer. I don't think I would have changed my decision, but I think I would have wanted to prepare better”

#### 3.2.1 Theme 1: Informational Needs

Informational needs include any information that parents require or perceive as important to make an informed medical decision.

*Sub‐Theme 1*: Many parents expressed the importance of learning the basic medical information and statistics regarding transplant (Table 2, Q1). This included the anatomy of the normal heart; medication names, indications, and side effects (Table 2, Q2); wait list timeline; and survival, complication, and retransplant statistics (Table 2, Q3). Several families were interested in understanding how the transplant would affect other organ systems (e.g., renal, gastrointestinal). They wanted to understand the transplant and VAD surgeries and the predicted recovery.

*Sub‐Theme 2*: Parents expressed the importance of connecting with like peers (other parents of children listed for or following heart transplant) to understand the practical aspects of life with a child who is waiting for or has undergone a heart transplant. While the medical team offers concrete facts, the intangible experience, the gravity of the impact on daily life, and practical advice were better appreciated through discussion with like peers (Table 2, Q4–Q6).

#### 3.2.2 Theme 2: Decision Making Perception

Decision making perception incorporates the degree to which families perceive there is a medical decision to be made and to what degree they desire agency in making the medical decision.

*Sub‐Theme 1*: For many parents, proceeding with transplant or VAD was a foregone conclusion given that the alternative was mortality of the child (Table 2, Q7). However, some wrestled with the decision before ultimately deciding to proceed. They considered the challenges and suffering their child had already experienced, the potential for suffering and complications during the wait list period, and the post‐transplant prognosis (Table 2, Q8 and Q9).

*Sub‐Theme 2*: The majority of parents wanted full responsibility for the decision whether or not to proceed with VAD or transplant (Table 2, Q10 and Q11). Some parents preferred a collaborative approach with the medical team (Table 2, Q12). Very few parents preferred the medical team to make the decision.

#### 3.2.3 Theme 3: Decision Factors

Decision factors include any outcomes or influences that impact one's medical decision making including outcomes of the decision itself or outside factors that influence one's decision.

*Sub‐Theme 1*: The most important outcome families sought for their child was survival and good quality of life (Table 2, Q13–Q15). Quality of life was by far the most often stated factor that drove decision making. Some recognized the time and treatment needed while awaiting and recovering from transplant may negatively affect quality of life. But ultimately, most felt the short‐term negative impact on quality of life was worthwhile for the long‐term survival and improved quality of life for months and years after transplant.

*Sub‐Theme 2*: Outside factors were highly relevant to the experience but ultimately for most parents had minimal impact on decision making. Families acknowledged the myriad challenges associated with the transplant process (Table 2, Q16). These included emotional distress, spousal and sibling relationship strain, financial and employment stress, and difficulty balancing responsibilities with their hospitalized child and responsibilities outside the hospital. Those who had to geographically relocate due to the transplant noted this was especially stressful. However, for many, these challenges were viewed as the unfortunate but necessary consequences rather than factors that should influence their decision making (Table 2, Q17 and Q18).

#### 3.2.4 Theme 4: Parental Distress

Parental distress encompasses the emotional turmoil experienced secondary to their child's illness and the realities of the pre‐ and post‐transplant medical care.

*Sub‐Theme*: While not specifically solicited in the conversation guide, parental distress was prominent in the participant responses. They expressed a desire for mental health support (Table 2, Q19). They emphasized this process is emotionally taxing (Table 2, Q20) and wished they would have been more prepared for the emotional burden (Table 2, Q21 and Q22).

### 3.3 Educational Tool and Decision Aid Preferences

Educational tool and decision aid preferences included features and formatting for these tools that would make them useful and accessible.

#### 3.3.1 Formatting

Parents expressed a preference for both digital (e.g., online) and physical formats (e.g., paper) to address diverse needs. Digital formats were appreciated for their on‐demand access, ability to accommodate customizable and interactive features, ease of sharing with family and friends, and the fact that they cannot be misplaced. Physical copies, on the other hand, were valued for their accessibility without requiring a device, practicality for referencing during conversations with the medical team, and the intangible comfort of having a physical resource to hold and use.

#### 3.3.2 Understandable

Parents emphasized the importance of easy data visualization, user‐friendly navigation, and guided education. Families strongly prefer videos and diagrams over text. They prefer information broken into categories to make initial learning less overwhelming and to facilitate easier referencing and recall later. To further simplify data presentation, families suggested a multi‐layered format of data presentation wherein more simplistic information is initially presented for a given topic with “click here for more” option for more details as needed/applicable for their child. They acknowledged that the emotional and physical exhaustion associated with the heart transplant process decreased their capacity to engage with complex material independently. Thus, they prefer access to resources for initial review followed by guided education with providers.

#### 3.3.3 Sharable

Participants noted the need for simplified, shareable resources for family and friends to foster understanding and reduce the burden of repeated explanations. Tools should include light, accessible explanations that balance the need for information with sensitivity, avoiding overwhelming or alarming extended family and other support persons.

#### 3.3.4 Customizable

Parents were interested in a customized journey map that can be updated over time to keep them updated on the child's status and frame decisions not only at the outset (implanting a VAD, listing for transplant) but also as the wait period unfolds, quality of life changes, and/or complications arise.

## 4 Discussion

Parents of children with advanced heart failure face overwhelming and challenging medical decision making and an uncertain and arduous journey both pre‐ and post‐transplant. This study explored their informational needs, medical decision making perception, and outcomes and factors that impacted their medical decision making on behalf of their child. We found that parents seek medical information and practical advice from healthcare providers and like‐peers (other parents of children listed for or following heart transplant). They value autonomy in deciding the best path for their child, driven by the child's survival and quality of life, while other factors (e.g., financial, logistical, emotional challenges) are seen as inevitable consequences rather than decision making factors. Studies exploring informational needs, decision perception, and decision impactors have been undertaken in other pediatric contexts (other solid organ transplants, bone marrow transplant, chronically ill and medically complex children); this study in the pediatric heart transplant population corroborates and augments existing literature surrounding patient education and decision making in this unique clinical context.

### 4.1 Informational Needs: Like‐Peers as Psychosocial and Educational Support

While it is intuitive that families are interested in standard medical information (medication regimen, complications, survival and re‐transplant statistics) when facing heart transplant, our data highlights like‐peers as a critical source of not only psychosocial support but also other information and perspective. Like‐peers offer timely, practical advice that could be difficult to anticipate by the medical team. Furthermore, families felt underinformed and unprepared for the emotional burden they would face and felt that conversations (structured or spontaneous) with like‐peers may offer better preparation, perspective, and support for the gravity and scope of the impact of a child's heart transplant. Finally, families may find benefit from deliberation about medical decision making not only with the medical team and their social supports (family and close friends) but also with a peer mentor; this warrants further consideration and study as to its impact on decision quality and satisfaction.

Literature in pediatric medicine, including in the setting of organ transplant and VAD, has suggested that patients and families benefit from exposure like peers prior to an intervention (transplant, VAD, etc.) to improve their understanding of the realities of the intervention. Studies of pediatric patients prior to lung transplantation and VAD placement described families' desire to meet a child who had undergone these procedures to improve their comprehension of the reality [18, 19]. Our study suggested families may benefit from such pre‐procedure exposure and also long‐term peer mentorship and education. Salmon et al. described parents of kidney transplant recipients appreciating a peer support network as a source of psychosocial support and education [20]. One study in pediatric heart transplant specifically described many benefits from a camp for children and families following heart transplant including peer support and collaborative learning and problem solving [21]. As a result of our findings, we strongly recommend pediatric heart transplant programs consider not only one‐time pre‐transplant peer engagement, but supporting a formal peer to peer mentor program or establishing long‐term formal peer to peer education forums in addition to the transplant education and psychosocial supports that already exist in standard transplant programs Alternatively, linking families to peer support groups via social media or other online venues could be considered. A study exploring educational needs of post heart transplant recipients and their families noted families placing high value on topics affecting daily life such as medication schedules and clinic visit frequency [22]. A long‐term peer to peer mentor relationship could be one of many ways to address these educational needs, particularly learning and navigating the practicalities of daily life after transplant.

### 4.2 Decision Making Perception: Strongly Support Autonomy

The desire for autonomous or shared decision making (as opposed to the medical team making decisions) in our population was consistent with previously reported literature in pediatric solid organ transplant, adolescents with advanced heart failure, and critical illness [9, 10, 23, 24]. In the adult population, proceeding with VAD support and heart transplant is concretely framed as a medical decision [25]. With the creation and dissemination of the iDECIDE VAD Pediatric decision aid [26], the pediatric heart failure community is also more concretely framing VAD and transplant for advanced heart failure as an opportunity for contemplation, values clarification, and decision. Our data highlighted that many families in our cohort benefit from the framing as a decision as opposed to a foregone conclusion (e.g., Table 2, Q8 and Q9). Families acknowledged particularly the suffering and suboptimal quality of life experienced by their child leading into the VAD/transplant decision as a reason for contemplation and genuine decision making. While most families at our institution (and all in our study) ultimately elected to proceed, they appreciated the empowerment to make the final decision. Interestingly, in a study of seven families being presented with VAD support, six felt there was no viable alternative whereas one family (whose child did not survive to transplant) expressed reservations in the wake of their decision [18]. While the sample size between our two studies remains small, our study offers clear evidence that many families contemplate the choice.

Importantly, there are many ways for providers to support families in complex medical decision making, such as offering an “open menu” of possibilities with discussion of risks and benefits or thoughtfully soliciting family values and making a recommendation that aligns with those values with a clear explanation of how this recommendation was reached [27]. Using a decision aid that incorporates values clarification, such as the iDECIDE VAD Pediatric decision aid [26], can provide structure for these challenging conversations. We did not explore preferences in how shared decision making would be undertaken; this warrants further investigation in this context.

Our study highlights that heart transplant listing and post‐transplant care have immeasurable impacts on the child and family (as highlighted in the iDECIDE VAD Pediatric decision aid). Families need, and indeed prefer, to be informed and allowed autonomy to choose that journey. That said, they were significantly burdened by the weight of the pre‐transplant decision‐making and challenges. Intensive emotional and psychosocial support should be offered as families are deliberating and subsequently navigating the challenging (even if “worth it”) consequences.

### 4.3 Decision Factors: Tension or Co‐Existence of Survival and Quality of Life

Survival and quality of life were by far the most important considerations when parents were deciding whether to proceed with heart transplant for their child. Interestingly, these patients experienced many things that many would anecdotally perceive as negative impactors on quality of life (e.g., prolonged hospital stay, prolonged intubation, multiple and painful surgeries/medical procedures, chronic feeding intolerance, lifelong medical care and medication reliance, complications impacting neurodevelopment). This raises several points of tension. First, by proceeding with transplant and the acknowledged acute suffering it may cause, families who highly value quality of life demonstrate a willingness to tolerate temporary low quality of life (pre/peri‐transplant) for long‐term improved quality of life (post‐transplant) and improved odds of survival (when compared to a palliative care approach to advanced heart failure). None of the families we engaged explicitly outlined this thought process and it warrants further exploration of how families harmonize quality of life not only intrinsically (i.e., what interventions or outcomes would constitute unacceptable quality of life?) but when factoring in modifiers such as duration and final outcomes (i.e., how long would you tolerate poor quality of life if the outcome is good quality of life and long‐term survival?). Importantly, few tools (none specifically in the context of pediatric heart transplant) exist [28] to help families explore the possibilities and their preferences for their child's quality of life incorporating the complexities of acute and chronic nature and long‐term outcomes. Such a tool could be beneficial to this population who places high value in both quality of life and survival to balance and better define their decisional priorities. Another source of tension is that there may be a difference in the perceived value of pre‐transplant care (particularly if prolonged and complicated) for families and medical providers depending on the value placed on acute suffering as opposed to long‐term survival and quality of life outcomes between the two groups. While this was not specifically explored in this study, it has been noted in multiple other pediatric critical care settings [29, 30]. This has the potential to lead to moral distress among medical staff and warrants further investigation.

### 4.4 Education and Decision Support Tools: Simple and Dynamic

With regard to educational tool and decision aid preferences, parents' preferences aligned with recommendations for high‐quality educational materials including the use of simple language, visual aids and educating in small segments [31]. Specifically, they were interested in educational videos to supplement handwritten or printed materials. Parents underscored the importance of providing educational resources for extended family and friends. This approach ensures that the family's support network is adequately informed and capable of offering support, while alleviating the parents from the burden of educating. The preference for a customizable, evolving journey map highlights the importance of providing parents with a dynamic decision aid that reflects the complex and fluctuating nature of the heart transplant process. Such a resource could help parents navigate not only the initial steps (VAD implant, listing for transplant), but also the decisions that emerge during the wait period. In clinical practice, integrating a personalized, real‐time journey map could support parents by offering clear, actionable updates and facilitating timely decision‐making in light of an evolving clinical course.

This study had several limitations that lend themselves to future work. Out of consideration for families in the midst of decision making, we elected to engage pre‐transplant families via interviews as opposed to focus groups; dual methods of engagement may have influenced themes elicited via focus groups or interviews. Given the homogeneity of the patient population at our institution, our study lacked diversity with regard to race, primary language, religious affiliation, and gender. It was also geographically limited to the Intermountain West region. Cultural differences across geographic regions, races, ethnicities, and religions as well as the parent's relationship to the child (mother vs. father) may impact the themes we explored. Education level likely impacts informational needs and desired level of information; the majority of our participants had some college education or more which may make their informational needs skewed compared to a population that reflects national trends. Furthermore, the majority of our participants were parents of young children who did not have the capacity to contribute to decision making. It would be interesting to explore similar themes with more parents of adolescent patients and with adolescent patients themselves to understand how their thoughts and opinions about their care might contribute to the family's medical decision making. Finally, we were unable to engage any families whose child had died while listed for or following heart transplant. It would be beneficial to explore similar questions with such families to understand how their perception and decision making compare to those in our study.

In conclusion, medical decision‐making for a child with heart failure is incredibly challenging. Parents desire basic medical information from their medical team but also more day‐to‐day practical advice that is primarily derived from the lived experiences of like‐peers. Parents appreciated the autonomy to choose the best path for their child and their family when contemplating heart transplant or mechanical support. The child's survival and quality of life were by far the most important factors that drove decision‐making. Other factors were acknowledged as significant stressors but viewed as inevitable consequences as opposed to drivers of decision‐making. Together, these findings underscore the importance of peer support groups, emphasize the need for social support to assist in navigation of external stressors, suggest value in the development of a decision support tool to assist families in solidifying and voicing their goals and values, and highlight the need for future research to explore discrepancies between parent and provider perceived quality of life and value of care in this population.

## Data Availability

The data that support the findings of this study are available on request from the corresponding author. The data are not publicly available due to privacy or ethical restrictions.
